# Physicochemical Properties of Hyaluronic Acid–Based Lubricant Eye Drops

**DOI:** 10.1167/tvst.8.6.2

**Published:** 2019-11-01

**Authors:** Pasquale Aragona, Peter A. Simmons, Hongpeng Wang, Tao Wang

**Affiliations:** 1 Department of Biomedical Science, Regional Referral Center for Ocular Surface Diseases, University of Messina, Messina, Italy; 2 Allergan plc, Irvine, CA, USA; 3 School of Optometry and Vision Science, Faculty of Science, University of New South Wales, Sydney, Australia

**Keywords:** dry eye, artificial tear, eye drop, hyaluronic acid, hyaluronate, glycosaminoglycan, viscosity

## Abstract

**Purpose:**

To assess the physicochemical properties of hyaluronic acid (HA)-based artificial tears.

**Methods:**

The average molecular weight (MW) and polydispersion index (PDI) of HA in 18 commercially available artificial tears were determined by light scattering/high-performance liquid chromatography. Osmolality, pH, viscosity, and sodium concentration were determined using an osmometer, pH meter, rheometer, and inductively coupled plasma mass spectrometer, respectively.

**Results:**

The MW of HA varied considerably between formulations. The PDI was >2.0 in two formulations (2.28 and 4.94), suggesting the presence of a copolymer and/or HA size variability. Three formulations exhibited viscosity exceeding the blur threshold at different shear rates. Viscosity at low shear rates was generally highest in formulations containing high-MW HA. Correlations were found between observed viscosity and a predictive/calculated value, except for four copolymer-containing formulations, and osmolality (range, 154–335 mOsm/kg) and sodium concentration (range, 22–183 mM), with two exceptions. Compared with organic osmolytes, adding sodium decreased viscosity, particularly at lower shear rates.

**Conclusions:**

In the context of the literature, our findings suggest that for most patients with dry eye disease, the ideal HA-based artificial tear should include high-MW HA with a low PDI and exhibit enhanced viscosity at low shear rate (without exceeding the blur threshold). The inclusion of synergistic copolymers and a low sodium concentration may increase viscosity, but whether any of these physicochemical properties or correlations can predict clinical efficacy will require further investigation.

**Translational Relevance:**

Understanding the properties of HA-based artificial tears will support the development of unique formulations that target specific ocular surface conditions.

## Introduction

Artificial tears are designed to supplement or substitute for normal tears and alleviate dry eye disease. Currently available formulations contain hydrophilic polymers such as hyaluronic acid (HA) and/or cellulose ethers that augment viscosity, improve retention time, and optimize ocular surface hydration and lubrication.^1^,^2^ HA is a strongly hydrophilic, naturally occurring, nonsulfated glycosaminoglycan composed of disaccharide units of glucuronic acid and N-acetyl-D-glucosamine; its molecular weight (MW) ranges from <100 to >1000 kDa and it is primarily located in the extracellular matrix, where it plays an important structural role.^3^ HA also provides viscoelasticity to biological fluids, such as the vitreous humor,^3^,^4^ and induces cellular signaling in a size-dependent manner^5^,^6^ by binding to CD44, a polymorphic glycoprotein receptor that is expressed ubiquitously^7^,^8^ and has been implicated in the hydration of the ocular surface, as well as regeneration of the corneal epithelium.^9^,^10^ Notably, CD44 is overexpressed in corneal/conjunctival cells where there is ocular surface damage.^11^

Preclinical and clinical studies have shown that artificial tears containing HA provide acute and long-term therapeutic benefits in dry eye disease, including enhancement of corneal epithelium healing, improvement of the ocular surface function, and protection/restoration of the morphology and distribution of goblet cells (which secrete mucins).^11^–^17^ These formulations, however, can vary in terms of HA concentration (0.1%^18^ to 0.5%^19^), MW (<100 to >1000 kDa^20^,^21^), presence of other polymers (e.g., carboxymethylcellulose [CMC]^22^), polydispersion index^23^–^26^ (PDI; reflecting uniformity of MW distribution), and osmolality/osmolarity.^27^–^30^ Formulations containing chemically modified (e.g., cross-linked, thiolated^31^,^32^) HA are also being investigated. All of these variations may, for example, impact clinical performance by affecting the solution viscosity^33^–^35^ and ability to resist deformation under shear stress.

Rheology assesses fluid flow and deformation under the influence of mechanical force.^36^ One type of rheological analysis measures changes in viscosity under changing shear rate, which may be used to model the behavior of fluids on the ocular surface in changing conditions of eye movements and blinking. Natural tears are categorized as non-Newtonian fluids because their viscosity depends on shear rate.^37^ When open, the eye benefits from a higher tear viscosity to prevent drainage and tear film break-up, whereas a lower tear viscosity during blinking prevents damage to the epithelial surface.^38^ To improve ocular comfort, reduce friction (which can lead to inflammation), and minimize vision blurring, the ideal artificial tear formulation should, thus, demonstrate reduced viscosity (or shear thinning) at high shear rates, such as occurs during blinking.

Artificial tear formulations also usually aim to mimic the osmolarity or osmolality of newly formed, natural tears (i.e., ∼304 mOsm/L^39^ or ∼304 mOsm/kg^40^), as tear hyperosmolarity has been shown to be deleterious to the ocular surface, triggering inflammation and exacerbating tear film instability.^41^ Some artificial tear formulations are specifically compounded to be hypotonic (e.g., Hyabak, Thealoz Duo, and Thealoz Duo Gel^42^), on the basis that this may help counteract the hyperosmolarity found in dry eye tear fluid.^41^,^43^,^44^ Regardless of target total osmolarity, most artificial tear formulations utilize standard electrolytes, principally NaCl, to adjust tonicity.^39^ Some formulations, however, include nonelectrolyte organic osmolytes, such as glycerin, other polyols, or amino acids, to provide formula tonicity. A number of these organic osmolytes have been demonstrated to have anti-inflammatory properties in various cell and animal models of dry eye.^45^–^47^

Manufacturers generally indicate the percentage of HA included in their formulations, but such partial information could be of limited value when trying to determine the best formulation for a patient with dry eye, as the MW and PDI of HA used, together with other physicochemical properties, can also contribute notably to the overall viscosity and clinical indications of a formulation. In fact, different characteristics of a tear substitute can be advantageous in different stages or types of dry eye.^48^ Although most formulations evaluated in this study are labeled for the relief of symptoms such as irritation, redness, burning, itching, and grittiness/foreign body sensation associated with dry eye caused by environmental stress (e.g., wind, air conditioning, drought, and smoke) and eye strain (from computer work), some are recommended to ensure protection during corneal repair processes (e.g., Hyalistil Bio^49^), whereas others are recommended for symptoms/signs of dry eye and/or ocular surface damage due to diseases, such as superficial keratitis, Sjögren's syndrome, or primary dry eye syndrome (e.g., Vismed Multi^50^).

To help better adapt artificial tears to different ocular surface conditions, this study assessed the MW and PDI of HA in marketed artificial tears, as well as the viscosity, pH, osmolality, and sodium concentration (main electrolyte contributing to tear osmolarity/osmolality^39^) of those formulations, and evaluated whether these physicochemical characteristics can affect clinical performance and predict efficacy.

## Materials and Methods

**Figure 1:**
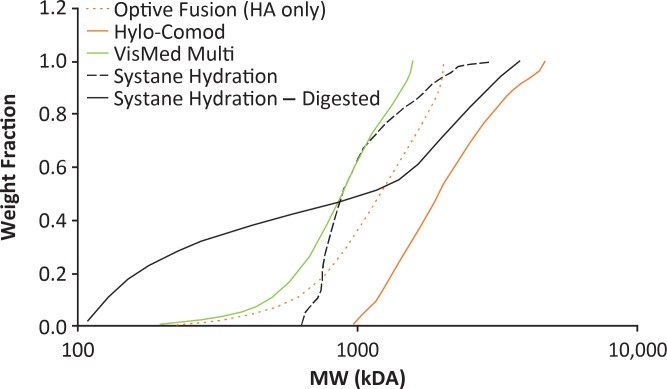
Typical output of a MW analysis of HA-based artificial tears based on size exclusion chromatography. The average MW of the overall polymer component of a given artificial tear formulation was measured on a high-performance liquid chromatograph, as described in the Materials and Methods. Examples of formulations containing high- (Hylo-Comod), medium- (Vismed Multi), and low- (Systane Hydration) MW HA are shown, compared with the HA component of the two Optive products. The increase in the average MW of Systane Hydration after hyaluronidase treatment suggests the presence of low-MW HA and predominance of a high-MW copolymer. HA, hyaluronic acid; MW, molecular weight.

Eighteen commercially available HA-based eye drops were evaluated (Table 1). The average MW and PDI of their overall polymer component were measured by light scattering on a high-performance liquid chromatograph (1200 series; Agilent Technologies, Santa Clara, CA) equipped with multiangle light scattering (Dawn Heleos II) and refractive index (Optilab T-rEX) detectors (Wyatt Technology, Santa Barbara, CA). Eye drop samples were diluted 1:3 (v/v) with standard phosphate-buffered saline, filtered through a 0.45-μm low protein binding membrane, and separated on a size exclusion column (300 mm long, 7.8-mm internal diameter; WTC-030S5; Wyatt Technology) by using phosphate-buffered saline as mobile phase (flow rate, 0.5 mL/min; run time, 50 min). Bovine serum albumin (A1900; Sigma-Aldrich, St. Louis, MO) was used as the MW and PDI reference standard. Six of these products (Artelac Rebalance, Blink Intensive Tears, Optive Fusion multidose [available in the United States as Refresh Repair] and unit dose, Systane Hydration, and Thealoz Duo Gel) contained additional polymers (Table 1) and were analyzed before and after hyaluronidase treatment (80 IU [SAGE Media, Trumbull, CT] for 1 mg of HA; 37°C, 2 hours, 60 rpm in a combined orbital/linear shaking water bath [OLS200; Grant Instruments, Cambridge, United Kingdom]) to determine their contribution to the MW. Hylo-Parin did not require hyaluronidase treatment, as the heparin copolymer (Table 1) does not significantly affect MW assessments. Figure 1 shows a typical MW by weight fraction analysis of formulations containing high- (>1000 kDa), medium- (500–1000 kDa), and low- (<500 kDa) MW HA, compared with the HA component of two Optive products.

**Table 1:** HA-Based Artificial Tear Formulations Evaluated in the Study (grouped by MW)

Trade Name	Copolymer	Manufacturer
***Hylo-Comod***	***−***	***Ursapharm Arzneimittel GmbH, Saarbrücken, Germany***
***Hylo-Forte***	***−***	***Scope Ophthalmics, Dublin, Ireland***
***Hylo-Parin***	***Heparin*^a^**	***Ursapharm Arzneimittel GmbH***
***Optive Fusion (multidose)***	***CMC (0.5%)***	***Allergan plc***
***Optive Fusion (unit dose)***	***CMC (0.5%)***	***Allergan plc***
**Vismed Multi**	**−**	**TRB Chemedica Int. SA, Geneva, Switzerland**
**Xailin HA**	**−**	**Nicox, Valbonne, France**
**Artelac Rebalance**	**PEG 8000 (0.5%)**	**Bausch + Lomb, New York, NY**
**Hylo-Vision HD**	**−**	**OmniVision GmbH, Puchheim, Germany**
**Blink Intensive Tears**	**PEG 400 (0.25%)**	**Abbott Pharmaceuticals, Abbott Park, IL (a Johnson & Johnson Company)**
**BLUyal OSD**	**−**	**Pharma Stulln GmbH, Stulln, Germany**
**Hyalistil Bio**	**−**	**SIFI SpA, Lavinaio, Italy**
**Artelac Splash**	**−**	**Bausch + Lomb**
*Zolag*	*−*	*Laboratorios Grin, Mexico D.F., Mexico (acquired by Lupin Limited, Mumbai, India)*
*Hyabak*	*−*	*Thea Pharmaceuticals, Keele, Newcastle-under-Lyme, UK*
*Systane Hydration*	*Hydroxypropyl-guar (NA); PEG 400 (0.4%)*	*Alcon, Fort Worth, TX*
*Thealoz Duo*	*−*	*Thea Pharmaceuticals*
*Thealoz Duo Gel*	*Carbomer (0.25%)*	*Thea Pharmaceuticals*

Artificial tears that include high-, medium-, and low-MW HA are highlighted in bold-italic, bold, and italic, respectively. NA, not available; PEG, polyethylene glycol.

a Does not significantly affect MW assessment.

Osmolality and pH were determined using a standard osmometer (model 2020; Advanced Instruments, Inc., Norwood, MA) and an Accumet Excel XL 15 pH/mV/temperature meter (Thermo Fisher Scientific, Hampton, NH), respectively. The sodium concentration of each eye drop formulation (diluted 1:99 in 1% nitric acid, v/v) was measured using inductively coupled plasma mass spectrometry (model 7700; Agilent Technologies) calibrated with environmental standards (1000 μg/mL Na^+^ and other metals; Agilent Technologies).

Rheological properties of undiluted samples were evaluated at 35°C on a DHR-3 rheometer (TA Instruments, New Castle, DE) by using a 60-mm, aluminum Peltier plate (0.51° cone angle geometry; 15-μm measuring gap); varying the shear rate from 1 s^−1^ to 10,000 s^−1^ allowed assessment of potential shear thinning or thickening behaviors characteristic of non-Newtonian fluids.^51^ In addition, a value predictive of the standard observed viscosity (i.e., viscosity typically reported by manufacturers and measured with a standard instrument, such as a Brookfield viscometer) of each formulation was calculated by multiplying the HA concentration (%) and average MW (prehyaluronidase treatment when applicable).

Potential correlations between observed viscosity and the aforementioned predictive value, as well as sodium concentration and osmolality, were investigated using linear regression analysis. The relationship between viscosity and osmolality was also evaluated in a formulation containing organic osmolytes (Optive Fusion unit dose), before and after substituting organic osmolytes with 0.55% NaCl.

## Results

### MW and PDI of HA-Based Artificial Tears

Although the two Optive products contain high-MW HA (1178 kDa), the average MW of their polymer component was 318 kDa before the hyaluronidase treatment, decreasing to 201 kDa afterwards, which is consistent with low-MW CMC being a major contributor to the overall MW of those formulations, as previously reported.^35^ Following hyaluronidase treatment, the average MW of the polymer component of Artelac Rebalance and Blink Intensive Tears decreased from 902 and 772 kDa, respectively, to <100 kDa, suggesting that the MW was primarily due to HA (as opposed to the polyethylene glycol [PEG] copolymer; Table 1). In contrast, the decrease in average MW of the polymer component of Thealoz Duo Gel after hyaluronidase treatment was minimal (i.e., 204 kDa to 193 kDa), suggesting that the formulation contained low-MW HA that contributed to the total MW similarly to the carbomer polymer. On the other hand, the average MW of the polymer component of Systane Hydration increased from 1334 kDa to 2233 kDa after treatment with hyaluronidase, suggesting the presence of low-MW HA ([2233 + x]/2 = 1334; x = 435 kDa) and predominance of the high-MW hydroxypropyl-guar polymer (Table 1). These findings, together with information in Table 1, indicate that the average MW of HA was >1000 kDa in five of the 18 artificial tear formulations analyzed: Hylo-Comod, Hylo-Forte, Hylo-Parin, and the two Optive products (Table 2). In eight others (Vismed Multi, Xailin HA, Artelac Rebalance, Hylo-Vision HD, Blink Intensive Tears, BLUyal OSD, Hyalistil Bio, and Artelac Splash), the average MW of HA ranged between 500 and 1000 kDa. In the last five formulations (Zolag, Hyabak, Systane Hydration, Thealoz Duo, and Thealoz Duo Gel), the average MW of HA was <500 kDa (Table 2).

**Table 2:** Average MW, PDI, Osmolality, and Sodium Concentration of 18 HA-Based Commercially Available Artificial Tear Solutions

Test Product	[HA]^a^ (%)	Average MW^b^ (kDa)	Standard Viscosity^c^ (cP)	PDI	pH	Osmolality (mOsm/kg)	Sodium (mM)
***High-MW HA (>1000 kDa)***							
*** Hylo-Comod***	***0.10***	***2026***	***13.2***	***2.28***	***7.21***	***280***	***149***
*** Hylo-Forte***	***0.20***	***1748***	***80.6***	***1.79***	***7.20***	***280***	***132***
*** Hylo-Parin***	***0.10***	***1428***	***9.1***	***1.12***	***7.13***	***280***	***155***
*** Optive Fusion (multidose)***	***0.10***	***318 (201 post-H'ase)*^d^**	***15.5***	***3.49***	***7.37***	***335***	***57***
*** HA component***		***1178***		***1.10***			
*** Optive Fusion (unit dose)***	***0.10***	***318 (201 post-H'ase)*^d^**	***14.8***	***3.49***	***7.37***	***276***	***52***
*** HA component***		***1178***		***1.10***			
**Medium-MW HA (500–1000 kDa)**							
** Vismed Multi**	**0.18**	**918**	**11.5**	**1.20**	**7.16**	**154**	**88**
** Xailin HA**	**0.20**	**914**	**11.1**	**1.20**	**6.94**	**240**	**157**
** Artelac Rebalance**	**0.15**	**902 (<100 post-H'ase)^e^**	**7.8**	**1.13**	**7.30**	**279**	**128**
** Hylo-Vision HD**	**0.10**	**851**	**3.3**	**1.16**	**6.95**	**294**	**183**
** Blink Intensive Tears**	**0.20**	**772 (<100 post-H'ase)^e^**	**10.0**	**1.06**	**7.29**	**178**	**22**
** BLUyal OSD**	**0.15**	**694**	**5.1**	**1.05**	**6.99**	**286**	**178**
** Hyalistil Bio**	**0.20**	**650**	**9.2**	**1.13**	**7.16**	**230**	**146**
** Artelac Splash**	**0.24**	**533**	**7.2**	**1.11**	**7.10**	**288**	**145**
*Low-MW HA (<500 kDa)*							
* Zolag*	*NA*	*327*	*9.9*	*1.11*	*7.29*	*287*	*142*
* Hyabak*	*0.15*	*248*	*2.5*	*1.07*	*7.12*	*211*	*117*
* Systane Hydration*	*0.15*	*1334 (2233 post-H'ase)*^f^	*4.7*	*1.44*	*7.90*	*280*	*121*
* Thealoz Duo*	*0.15*	*220*	*2.8*	*4.94*	*7.09*	*209*	*56*
* Thealoz Duo Gel*	*0.15*	*204 (193 post-H'ase)*^g^	*2034.4*	*1.64*	*7.07*	*210*	*28*

Each property was assessed as described in the Materials and Methods. The MW of formulations containing one or more copolymer(s) was determined before and after treatment with hyaluronidase to evaluate the contribution of HA to the overall/average MW of the polymer component. Artificial tears that include high-, medium-, and low-MW HA are highlighted in bold-italic, bold, and italic, respectively. [HA], HA concentration; H'ase, hyaluronidase.

a Per the manufacturer.

b Average MW of all polymers included in the formulation, except where indicated.

c Assessed at 3 s^–1^ (i.e., low shear rate).

d Along with a posthyaluronidase PDI of 2.32, this suggests a significant contribution of the CMC (low MW) and HA (high MW) components. Considering information available for the HA (above) and CMC^35^ components of the two Optive products, this finding is consistent with low-MW CMC being a major contributor to the overall MW of those formulations, as previously reported.^35^

e Indicates that the average MW measured prior to digestion was primarily due to HA.

f Along with a posthyaluronidase PDI of 1.09, this suggests the presence of low-MW HA and predominance of high-MW hydroxypropyl-guar polymers, as polyethylene glycol 400 is a low-MW polymer.

g Along with a posthyaluronidase PDI of 1.35, this suggests that the formulation contained low-MW HA that contributed to the total/average MW similarly to the carbomer polymer.

Overall, the PDI of HA ranged from 1.06 to 4.94 and was ≤1.79 in 16 formulations (Table 2), including those with a known copolymer (see Table 1). The other two, namely, Hylo-Comod and Thealoz Duo, had a PDI of >2.0, suggesting the presence of an additional polymer (e.g., poloxamer 188) and/or a wide range of HA polymer size (possibly due to raw material selection and/or the manufacturing method).

### Rheological Analysis

**Figure 2:**
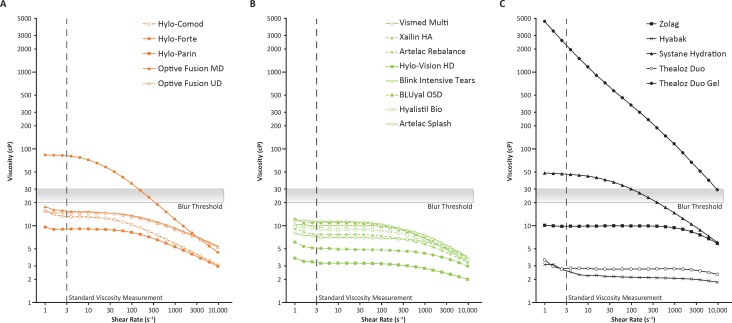
Rheological properties of artificial tears containing high-MW HA (orange) (A), medium-MW HA (green) (B), and low-MW HA (black) (C). Viscosity was evaluated as described in the Materials and Methods, varying the shear rate from 1 s^−1^ to 10,000 s^−1^ to allow assessment of potential shear-thinning or shear-thickening behaviors. The blur threshold (20–30 cP) is based on published study results showing that patients experienced a lesser degree (or amount) of visual disturbance with Optive Fusion (10–15 cP, containing 0.1% HA) than with a similar formulation containing 0.15% HA (35–40 cP).^52^ HA, hyaluronic acid; MD, multidose; MW, molecular weight; UD, unit dose.

Although all formulations exhibited shear thinning behavior characteristic of non-Newtonian fluids, viscosity was found to vary widely (Fig. 2^52^). Notably, two formulations demonstrated viscosity that exceeded the blur threshold at low shear rates: Systane Hydration (which combines 0.15% low-MW HA with hydroxypropyl-guar) and Hylo-Forte (which contains 0.2% high-MW HA). In addition, Thealoz Duo gel (which includes 0.15% low-MW HA along with carbomer) demonstrated viscosity above the blur threshold at all shear rates tested. The blur threshold (20–30 cP) was based on a study showing that patients experienced less visual disturbance with Optive Fusion (10–15 cP) than with a similar formulation containing 0.15% HA (35–40 cP) instead of 0.1%.^52^ Among the other 15 products, viscosity at low shear rates was found to be highest in formulations containing low concentrations of high-MW HA (i.e., the two Optive products and Hylo-Comod), with the exception of Hylo-Parin. Hylo-Vision HD, Thealoz Duo, and Hyabak exhibited the lowest viscosity at both low and high shear rates (Fig. 2).

**Figure 3:**
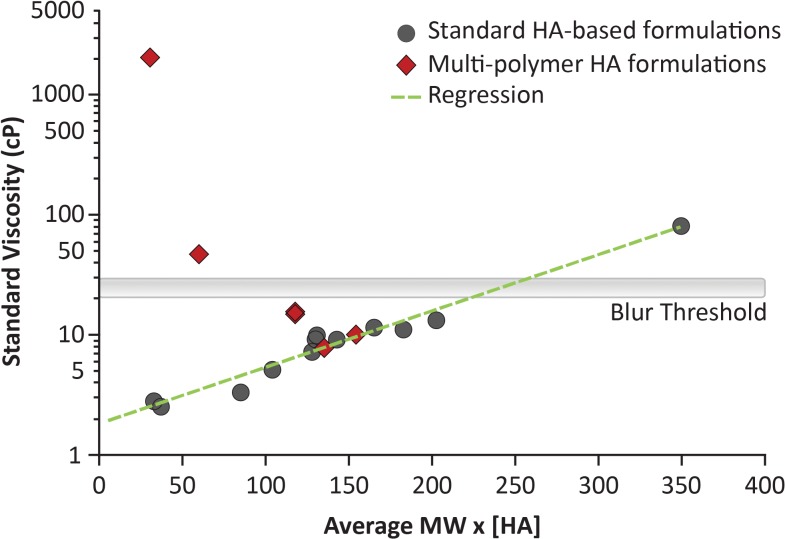
Standard observed viscosity as a function of the calculated value predictive of viscosity. The calculated value predictive of the standard observed viscosity (typically reported by manufacturers and measured with a standard instrument, such as a Brookfield viscometer) of each formulation was determined by multiplying the HA concentration (%) and average MW of the overall polymer component (prehyaluronidase treatment when applicable). Correlation is indicated by the green, dashed line; no attempt was made to fit the distal data points (four). The blur threshold (20–30 cP) is defined in Figure 2. HA, hyaluronic acid; MW, molecular weight.

Manufacturers typically report viscosity as measured with a standard instrument, such as a Brookfield viscometer, of which values are approximately equivalent to rheological viscosity assessed at a shear rate of 3 s^−1^. On the other hand, viscosity of HA-based formulations may be predicted by multiplying the MW and concentration of HA. Figure 3 shows that the calculated values correlated with the standard observed viscosity for all formulations, except Thealoz Duo Gel, Systane Hydration, and the Optive products, for which the observed values were higher than predicted, due to the presence of additional polymers. Notably, heparin, PEG 8000, and PEG 400 did not appear to negatively affect the aforementioned correlation when used as copolymers in Hylo-Parin, Artelac Rebalance, and Blink Intensive Tears, respectively.

### pH, Osmolality, and Sodium Concentration

**Figure 4:**
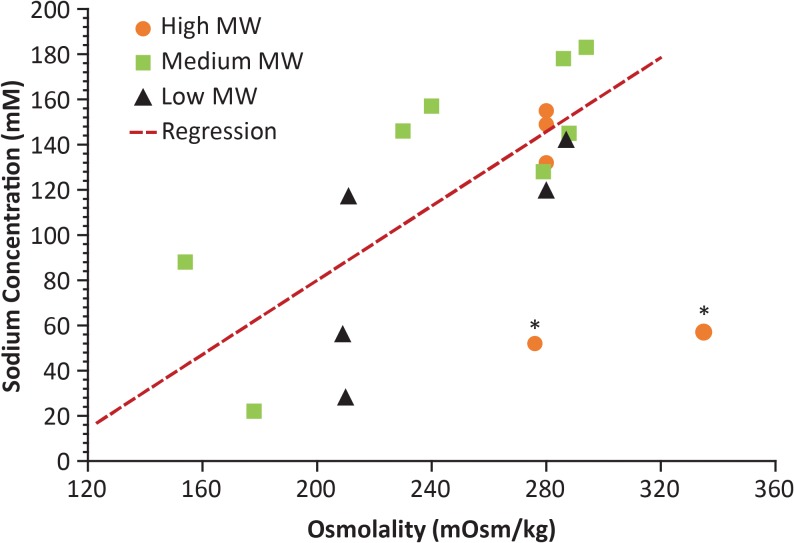
Relationship between sodium concentration and total osmolality. Correlation is indicated by the red, dashed line; no attempt was made to fit the distal data points (marked by an asterisk). * These two products have relatively high amounts of organic osmolytes. MW, molecular weight.

All formulations tested exhibited a pH that was close to neutral^53^ (Table 2). Their osmolality and sodium concentration ranged from 154 to 335 mOsm/kg and 22 to 183 mM, respectively, and a positive correlation was observed between these variables for all formulations, except the two Optive products that combined relatively high osmolality and low sodium concentration, compared with the other formulations (Fig. 4; Table 2).

**Figure 5:**
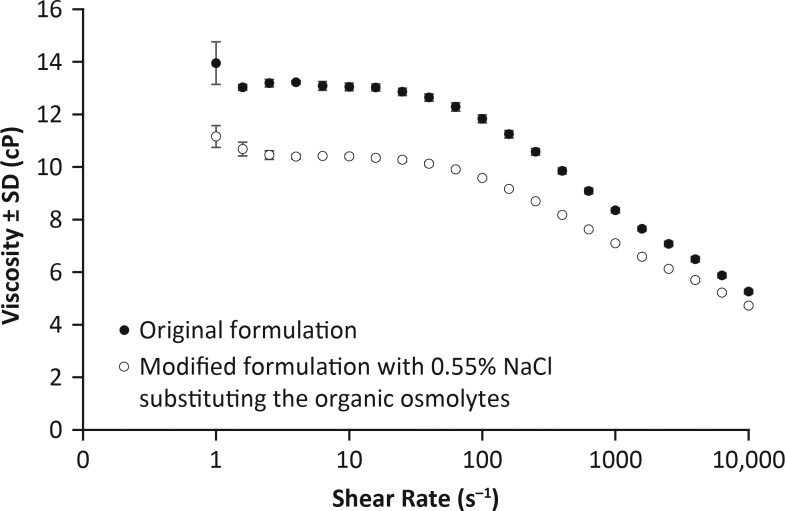
Effects of sodium versus osmolytes on viscosity. Viscosity was measured as described in the Materials and Methods by using the nonpreserved Optive product before and after substituting the osmolytes with 0.55% NaCl. The shear rate was varied from 1 s^−1^ to 10,000 s^−1^ to allow assessment of potential shear-thinning or shear-thickening behaviors. Total osmolality was 276 mOsm/kg in both formulations. P = 1.95 × 10^−10^, based on a paired Student's t-test comparing the rheology data before and after substituting the osmolytes with NaCl. Standard deviation (SD) values that are not visible are smaller than the data points.

To further explore the effects of sodium concentration on the properties of artificial tears, viscosity was assessed in the nonpreserved Optive product before and after substituting the organic osmolytes (glycerin, L-carnitine, and erythritol) contained in the formula with 0.55% NaCl (Fig. 5; Table 3). Results demonstrated that even though total osmolality was 276 mOsm/kg in each formulation, osmolytes and NaCl have statistically significantly different effects on viscosity, suggesting a relationship between osmolality/sodium concentration and viscosity.

**Table 3:** Decrease in Viscosity Observed After Substituting the Organic Osmolytes With NaCl, Keeping Total Osmolality at 276 mOsm/kg in Both Formulations

Nonpreserved Optive Product	Viscosity (cP) at the Indicated Shear Rate				
	1 s^−1^	10 s^−1^	100 s^−1^	1000 s^−1^	10,000 s^−1^
Original formulation	14.0	13.1	11.8	8.4	5.3
Modified formulation with NaCl substituting the organic osmolytes	11.2*	10.4*	9.6*	7.1*	4.7*
Viscosity decrease (%)	20	20	19	15	10

* *P* < 2 × 10^−10^ (compared with the original formulation).

## Discussion

Overall, this study showed that there is substantial variability among artificial tear formulations in terms of concentration, MW, and PDI of HA, as well as osmolality and sodium concentration of the formulation. Considering that increased viscosity at low shear rates is beneficial to improve eye drop retention and optimize hydration/protection of the ocular surface between blinks^2^,^54^ and that reduced viscosity at high shear rates is desirable to improve ocular comfort (and possibly friction-related inflammation) during blinking, the effects of these physicochemical characteristics on the overall viscosity of each formulation are expected to influence their clinical performance. Artificial tears that exhibit low viscosity at low shear rates may indeed be more susceptible to rapid drainage or evaporation and preferred in cases where a thicker mucus secretion or the need to increase tear clearance is present, whereas blurred vision may occur if viscosity is too high (due to shear thickening or resistance to shear thinning). In contrast, the pH of all formulations (6.94–7.37) was well within the range previously shown not to affect the viscosity of HA (6.5–8.0)^55^ and should, thus, have a negligible impact on clinical performance.

In a monodisperse polymer sample, all molecules have the same MW and the PDI is 1.0.^56^ The fact that the PDI of the artificial tears tested varied from 1.06 to 4.94 is clinically relevant given that the physiological role of HA has been shown to depend on its MW.^6^ In particular, HA plays a key role in inflammation (which has been identified as having a causal etiologic role in dry eye^57^), with high- and low-MW HA acting as anti- and proinflammatory mediators, respectively.^58^–^60^ This suggests that an artificial tear containing higher-MW HA characterized by a low PDI (e.g., Hylo-Parin and the Optive products) might have a greater therapeutic potential, providing hydration, lubrication, and anti-inflammatory properties to protect the ocular surface. Of the two products with a PDI >2.0 and no reported copolymer, namely, Hylo-Comod and Thealoz Duo, the former is characterized by a high-MW HA and high viscosity at low shear rates (while remaining under the blur threshold), compared with a low-MW HA and low viscosity at low shear rates for the latter. It would, thus, be informative to compare their clinical performance, including other formulations as well, to determine whether these characteristics can be used as predictive factors for their specific clinical indication.

Among the 15 formulations with viscosity below the blur threshold at all shear rates, the shear thinning behavior of those consisting of high-MW HA should ensure sufficient viscosity between eye blinks to prevent drainage/evaporation and optimize residency time, while reducing friction on the ocular surface during blinks (and potentially inflammation). As might be expected, the calculated value predictive of viscosity (determined by multiplying the concentration [%] and MW of HA) underestimated the observed viscosity of formulations containing the large copolymers CMC, hydroxypropyl-guar, and carbomer. In contrast, the presence of heparin, PEG 8000, and PEG 400 did not affect the calculated value predictive of viscosity, possibly because their MWs (heparin, 12–15 kDa; PEG 8000, 8 kDa; PEG 400, 0.4 kDa) are comparatively much lower than those of HA and the other copolymers.

Polymers such as CMC (anionic and linear) can increase the molecular entanglement of HA,^35^ which, in turn, increases viscosity in a synergistic fashion^35^ to improve clinical performance of artificial tears, as demonstrated in a study showing that a combination of HA and CMC improved tear film stability and ocular surface symptoms of dry eye following cataract surgery.^61^ While hydroxypropyl-guar is cationic and forms random coils, carbomer is anionic and can exist in both linear and coiled conformations; their interaction with HA may, thus, be different, compared with CMC. Nonetheless, artificial tears containing copolymers might be preferred by patients with dry eye, but whether the presence of a copolymer may be predictive of clinical efficacy will require further investigation.

Our findings indicate that the osmolality and sodium concentration of the formulations tested varied by over two- and eight-fold, respectively. There was, however, an overall positive correlation between osmolality and sodium concentration, except for formulations with relatively high osmolality and low sodium content, consistent with the inclusion of additional organic osmolytes. Regardless, the observed correlation does not allow us to conclude as to whether it could be predictive of clinical efficacy. It should also be noted that the list of variables assessed in this study was not exhaustive and there are other physicochemical properties that can potentially influence the clinical performance of artificial tears, including surface tension, which can, in turn, vary with the concentration of other ingredients (e.g., preservatives, surfactants, or emulsifying agents). It would, thus, be informative to evaluate such properties and other rheological analyses in future studies.

In summary, our data (in the context of the literature) suggest that, among artificial tear formulations containing HA, there is great variability in the characteristics of HA. This variability makes it possible to choose the ideal HA-based artificial tear for a patient's clinical condition. A high-MW HA with a low PDI and higher viscosity at low shear rate (without exceeding the 20- to 30-cP blur threshold) should, for example, be preferred when patients present with high levels of ocular surface inflammation and epithelial damage. The presence of additional synergistic polymers and a low sodium concentration may also increase viscosity and performance and, thus, be beneficial. The initial objectives of treatment with artificial tears are to relieve symptoms of discomfort, heal the epithelium, and reduce inflammation, followed by efforts to maintain the tear film quality. The optimal formulation should, thus, be reevaluated as/if the ocular surface changes during follow-up.^48^ For instance, higher viscosity products may be desirable to treat ocular surface damage, whereas lower viscosity products may be preferred to maintain tear film stability, but a formulation that can achieve both is likely to be preferred by patients.
